# Small Airways Disease in Patients With COPD: A Question-and-Answer Approach for Everyday Clinical Practice

**DOI:** 10.1016/j.chest.2025.07.002

**Published:** 2025-07-16

**Authors:** Dimitrios Toumpanakis, Youlim Kim, Omar S. Usmani

**Affiliations:** a 2nd Department of Critical Care Medicine, “Attikon” University Hospital, Medical School, National and Kapodistrian University of Athens, Athens, Greece; b Division of Pulmonary and Allergy, Department of Internal Medicine, Konkuk University Medical Center, Konkuk University School of Medicine, Seoul, Korea; c National Heart and Lung Institute, Imperial College London and Royal Brompton Hospital, London, England

**Keywords:** clinical practice, COPD, oscillometry, patient-reported outcomes, small airways

## Abstract

**Topic Importance:**

Small airways are recognized as the main site of disease progression and airflow limitation in patients with COPD. Whereas conventional lung function testing, for example spirometry, is nonspecific to small airways disease (SAD), the advent and wider availability of techniques sensitive to SAD, such as oscillometry, has improved our understanding of the clinical importance of small airways dysfunction. Despite this progress, a gap between the recent advances in knowledge of SAD and its implementation in daily clinical practice remains. We aimed to answer key questions that would allow practitioners (eg, family doctors, internists, pulmonologists) to introduce oscillometry into their clinical practice.

**Review Findings:**

COPD pathogenesis is characterized by SAD, with an increasing prevalence with more advanced disease. Evaluation of small airways dysfunction with sensitive techniques (eg, oscillometry, nitrogen washout) contributes to early disease detection and plays a significant role in almost every aspect of disease assessment, including confirmation of diagnosis, functional severity grading, and monitoring of lung function decline. Moreover, small airways dysfunction shows equivalent or even better correlation with patient-reported outcomes, including symptoms, quality of life, and exacerbations, compared with conventional lung function testing. This suggests a role for small airways assessment as a treatable trait in COPD to target and monitor therapeutic interventions.

**Summary:**

Accumulating evidence and recent advances have delineated the role of small airways assessment in COPD and warrant its implementation in the management plan of patients with COPD in daily clinical practice.

Small airways (those with an internal diameter of < 2 mm) long have been recognized as the main physiologic site of airflow limitation in patients with COPD.^1^ Pathologically, advances in molecular biology have elucidated the underlying mechanistic processes in small airways disease (SAD), present even in mild disease (an overview of the immunopathologic features of SAD is found in the online data supplement). Structurally sensitive lung imaging techniques have enhanced visualization and augmented our understanding of the role of SAD in COPD.^2^

Yet, conventional lung function testing in the clinic, that is, spirometry, is limited in assessing SAD. However, the increased usefulness and validation of lung mechanics testing, mainly oscillometry, has expanded our understanding of SAD and its role in early manifestation, disease progression, and association with symptoms and quality of life (QoL) in patients with COPD to become a physiologic asset in the clinic.^3^ This structured question-and-answer review translates the gap between the recent advances in specialized knowledge of SAD to its pragmatic and relevant application in our everyday clinical practice with patients with COPD for family practitioners, internists, and pulmonologists.

## Literature Search

For our aim, we searched articles in PubMed and Google Scholar using keywords, including *small airways*, *COPD*, *oscillometry*, *symptoms*, and *outcomes*, focusing on studies exploring the clinical usefulness of small airways assessment in COPD. A more detailed description of our methodology is presented in e-Appendix 1.

## Evidence Review

### How Common Is SAD?

The small airways are considered to be the primary site of disease progression in COPD, and a variety of techniques have been used to detect dysfunction in this lung region (Table 1). A recent large population-based study estimated the prevalence of SAD at 43.5%, based on spirometric parameters (forced expiratory flow between 25% and 75% of vital capacity [maximum midexpiratory flow, or FEF_25%-75%_], forced expiratory flow at 50% of vital capacity, and FEF at 75% of vital capacity), with major risk factors including, smoking, air pollution, and obesity.^4^ Crisafulli et al^5^ assessed small airways dysfunction in patients with COPD using oscillometry and showed an overall prevalence of approximately 70%, with increased prevalence associated with advancing Global Initiative for Chronic Lung Disease (GOLD) staging severity (based on FEV_1_ after bronchodilation), from 51% in patients with GOLD stage 2 disease (50% ≤ FEV_1_ < 80% predicted) to 95% prevalence in patients with GOLD stage 4 disease (FEV_1_ < 30% predicted).^6^

**Table 1:** Prevalence of Small Airways Dysfunction in the General Population, in People Who Smoke Who Have Preserved Spirometry Findings, and in Patients With COPD

Participants	Nomenclature and Definition	Prevalence	Reference
Overall population			
BOLD study (n = 28,604)	Small airways obstruction: spirometry findings (FEF_25%-75%_ < LLN or FEV_3_ to FVC ratio < LLN)	5%-34% (FEF_25%-75%_), 5%-31% (FEV_3_ to FVC ratio), depending on geographical distribution	7
China Pulmonary Health Study Group (n = 50,479)	Small airways dysfunction: spirometry findings (at least 2 of FEF_25%-75%_, FEF_50%_, or FEF_75%_ < 65% predicted)	43.5%	4
UK Biobank (n = 252,877)	Small airways obstruction: spirometry findings (FEV_3_ to FVC ratio < LLN)	23.6% (9.5% isolated small airways obstruction)	8
Participants with smoking history			
COPDGene cohort (n = 4,386)	SAD: spirometry findings (FEV_3_ to FEV_6_ ratio < LLN)	15.4% of patients with preserved FEV_1_ to FVC ratio	9
COPDGene cohort (n = 1,508)	fSAD: imaging findings (parametric response mapping, extent of fSAD)^a^	Preserved FEV_1_ to FVC ratio (n = 751), 12.4%); GOLD stage 1 (n = 150), 22.2%; GOLD stage 2 (n = 356), 26.6%; GOLD stage 3 (n = 192), 36.3%; and GOLD stage 4 (n = 59), 39.2%	2
Patients with COPD			
SPIROMICS cohort (n = 1,105 with mean FEV_1_ of 63.27% predicted)	fSAD: imaging (parametric response mapping, extent of fSAD)^a^	25%	10
ECLIPSE cohort (n = 2,054)	SAD: not defined, suggested by oscillometry (abnormal R_5_ – R_20_ or Ax, ie, outside of 90% CIs of nonsmoking control group)	Control participants who smoke: R_5_ – R_20_, 6%; Ax, 5% Patients with COPD: R_5_ – R_20_, 60%, Ax, 71% Patients with GOLD stage 2 disease (n = 915): Ax, 51.7% Patients with GOLD stage 3 disease (n = 859): Ax, 84.5% Patients with GOLD stage 4 disease (n = 277): Ax, 94.9%	6
Crisafulli et al^5^ (n = 202 with mean FEV_1_ of 55% predicted)	Small airways dysfunction: oscillometry (R_5_ – R_20_ ≥ 0.07 kPa/s/L)	73.8%	5

Ax = area under the curve of reactance from the lowest frequency measured; BOLD = Burden of Obstructive Lung Disease; COPDGene = Genetic Epidemiology of Chronic Obstructive Pulmonary Disease; ECLIPSE = Evaluation of COPD Longitudinally to Identify Predictive Surrogate Endpoints; FEF_50%_ = forced expiratory flow at 50% of vital capacity; FEF_75%_ = forced expiratory flow at 75% of vital capacity; FEF_25%-75%_ = forced expiratory flow between 25% and 75% of vital capacity (maximum midexpiratory flow); fSAD = functional small airways disease; GOLD = Global Initiative for Chronic Obstructive Lung Disease; LLN = lower limit of normal; R_5_ = total resistance at 5 Hz; R_20_ = total resistance at 20 Hz; SAD = small airways disease; SPIROMICS = Subpopulations and Intermediate Outcome Measures in COPD Study.

a For imaging studies, the average extent of fSAD on CT scans is presented, and not the prevalence itself.

Thus, SAD is a common characteristic of COPD pathophysiologic features, with increasing prevalence at more severe disease. The increased prevalence of SAD justifies the active detection and grading of SAD severity in daily clinical practice, which, as discussed herein, offers significant information for COPD diagnosis and assessment.

### How Can We Assess SAD in Everyday Practice?

#### Spirometry

The most commonly used indices of the FEV_1_, FVC, and FEV_1_ to FVC ratio are insensitive to small airways obstruction, whereas current European Respiratory Society and American Thoracic Society technical standards do not support the use of spirometry to identify SAD.^11^ Traditionally, FEF_25%-75%_ is considered a marker of SAD, although it is nonspecific for small airways, highly variable and poorly reproducible,^12^ and of minimal advantage when added to FEV_1_, FVC, and FEV_1_ to FVC ratio as a composite index.^13^ However, spirometry does allow assessment of emptying of the small airways at end expiration through a reduced FEV_3_ to FEV_6_ ratio,^7^^,^^9^ and an increased difference between slow vital capacity and FVC.^12^ Indeed, in a recent study in those with a current and former smoking history without spirometric obstruction, a reduced FEV_3_ to FEV_6_ ratio was associated with increased functional SAD on lung imaging, lower FEV_1_, and a faster progression to COPD.^14^

#### Body Plethysmography

Similar to spirometry, assessment of lung volumes by body plethysmography can provide indirect evidence of SAD through residual volume to total lung capacity ratio denoting the presence of gas trapping.^12^

#### Oscillometry

**Figure 1:**
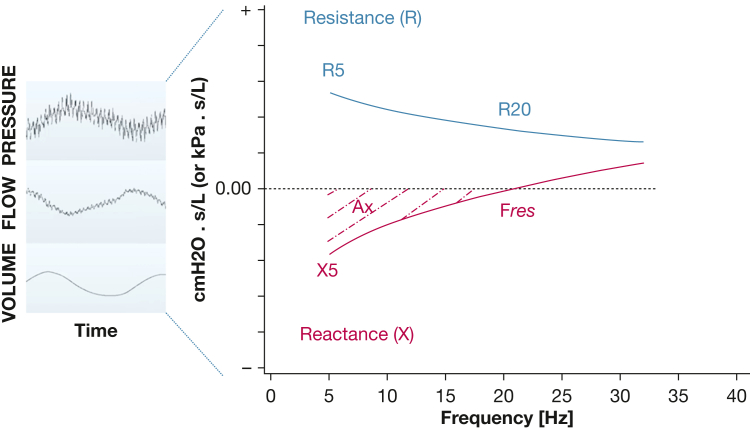
What is oscillometry? In 1956, Otis et al described the physics of the mechanical factors in distribution of pulmonary ventilation, setting the theoretical base of oscillometry.^22^ During the test, a signal of varying frequencies (either as impulses [impulse oscillometry]) or pseudorandom noise (forced oscillation technique) is applied at the mouth while the patient is breathing quietly, so no forced lung maneuver is performed, unlike spirometry. As shown, pressure and flow through the airways are measured by the oscillometer and the indices measured are R and X, which is the sum of elastance and inertance, that is, the opposition to changes in flow rate.^16^ Both R and X are elements of the impedance of the respiratory system calculated as a function of the frequency of oscillation. Anywhere a significant flow is found in the system will contribute to the resistance. The frequency dependence of resistance is the result of the distribution of time constants (resistance × compliance) of the smaller airways, thus reflecting the heterogeneity of the airway tree. Higher frequencies are more impeded by higher time constants than the lower frequencies. In healthy adult lungs, we should expect the frequency dependence to be close to 0, but with raises in small airways dysfunction (measured resistance increases at lower frequencies). The frequency dependence is arbitrarily, and for historical reasons, calculated between 5 Hz and either 19 Hz or 20 Hz; thus, R_5_ – R_20_ or R_5_ – R_20_, respectively, is a marker of small airways disease (SAD), whereas R_5_ and R_20_ are considered markers of total and central airway resistance, respectively. Modern devices typically use 19 Hz because this is a prime number relative to 5 Hz and will not cause harmonic interference, as in some older devices. Reactance at the lower frequencies (eg, X_5_) mainly reflects the elastic properties of the respiratory system, and F_res_ is the frequency where elastic and inertial properties are equal but opposing, and X is 0. As discussed in the text, X_5_ is also related strongly to the lung volume, and if an airway has been derecruited, no flow exists; hence, the impedance will be explained by the change in volume. Thus, the tissue contribution to the reactance is small in the frequency range typically used in conscious humans. Derecruitment (small airways closure) is by far the biggest cause of increased elastance. Thus, Ax (frequency dependence of reactance, ie, the area under the curve of reactance from the lowest frequency measured, eg, 5 Hz, to the F_res_) also serves as a marker of SAD in COPD. However, we must acknowledge that both R_5_ – R_19_ or R_5_ – R_20_ and Ax also can be affected by other sources of ventilation inhomogeneity, such as heterogenous time constants resulting from emphysema and changes in tissue viscoelasticity,^15^ although the contribution from tissue stiffness and viscoelasticity is small in the frequency range used in humans. For a detailed description of oscillometry, the interested reader is referred to available textbooks in the field.^17^^,^^18^ Similar to so-called conventional lung function tests,^11^ modern software allows the interpretation of oscillometry parameters based on *z* scores from published reference values.^25^ A recent systematic review noted variability among published reference equations^19^ because of variations in methodology and different devices. Indeed, different commercially available devices may lead to variability in results, especially in higher impedance values,^20^ and further work is needed toward generating the universal adoption of oscillometric reference equations. Using ethnically and racially neutral reference equations with oscillometry also is not explored. For this reason, interpretation based on cutoff values for oscillometric indices also has been investigated, where for R_5_ – R_20_ and Ax, values of 0.07 to 0.1 kPa/s/L and 1.0 kPa/L, respectively, are widely accepted as the upper limit of normal, used to detect SAD.^21^Ax = area under the curve of reactance from the lowest frequency measured; F_res_ = resonant frequency; R = resistance; R_5_ = total resistance at 5 Hz; R_19_ = total resistance at 19 Hz; R_20_ = total resistance at 20 Hz; X = reactance; X_5_ = reactance at 5 Hz.

Oscillometry is an increasingly important, noninvasive, reproducible physiologic tool for assessing SAD.^15^, ^16^, ^17^, ^18^, ^19^, ^20^, ^21^ During the test, a signal of varying frequencies is applied at the mouth while the patient is breathing quietly, so no forced lung maneuvre is performed, unlike spirometry. Oscillometry enables the measurement of the resistance (R) and the reactance of the respiratory system, the latter reflecting mainly elasticity at the lower frequencies (a more detailed description of oscillometry is presented in Fig 1).^16^ When present, SAD results in a frequency dependence of resistance,^22^ that is, resistance values increase at lower frequencies (eg, at 5 Hz); thus, the difference of total resistance at 5 Hz (R_5_) from total resistance at 20 Hz (R_20_) or from total resistance at 19 Hz (R_19_; R_5_ – R_19_ or R_5_ – R_20_, respectively) is used as a marker of small airways resistance, whereas R_20_ reflects central airways resistance (Fig 1).^16^ Interestingly, in patients with COPD, reactance also is worsened (becomes more negative, and thus lungs seem more to be stiff) because of SAD and premature airway closure that reduce communicating lung volume, thus reactance at 5 Hz (X_5_) also may indicate the presence of small airways pathologic features.^23^ Alterations in small airways caliber also may result in increased frequency dependence of reactance.^16^ Hence the area under the curve of reactance from the lowest frequency measured (Ax; eg, 5 Hz) to the resonant frequency also serves as a marker of SAD in COPD. The dependence of elastance to lung volume was supported further in a study recruiting patients with COPD and using hyperpolarized ^3^He MRI, where ventilation (lung volume) defects were correlated significantly to reactance indices, including reactance at 5 Hz and Ax.^24^

#### Inert Gas Washout Measurement

Mostly nitrogen washout using a single-breath or multibreath maneuver has been used and shown to be a sensitive technique to assess ventilation inhomogeneity and SAD.^12^ In a landmark study by Cosio et al,^26^ closing volume, that is, the lung volume at which the small airways start to collapse, especially in dependent areas, and the slope of phase 3 single-breath nitrogen washout were deranged in a group of patients who smoked and showed pathologic evidence of SAD, despite normal spirometry findings. Multibreath nitrogen washout allows a more detailed structure-function analysis in which ventilation inhomogeneity can be assessed either globally by the lung clearance index or more accurately partitioned into proximal (conductive airways inhomogeneity) or peripheral (acinar zone inhomogeneity, ie, peripheral airways, alveoli, or both) sources. Data show that in those who smoke, increased conductive airways and acinar zone inhomogeneity can be detected by multibreath nitrogen washout at early stages of disease, when SAD predominates,^27^ although in more advanced disease, ventilation inhomogeneity also is affected by emphysematous parenchymal destruction.

#### Chest CT Imaging

Although the small airways are below the spatial resolution of chest CT imaging, the presence of gas trapping in expiratory CT scans is an indirect marker of SAD. Recently, novel imaging algorithms have enabled the detection of SAD,^2^ giving confidence to the potentially routine role of CT imaging in assessing SAD in patients with COPD for diagnostic and monitoring purposes.^28^ By combining both inspiratory and expiratory CT images and applying attenuation cutoffs for emphysema and gas trapping (parametric response mapping), an imaging marker of functional SAD has been defined that correlates with FEV_1_ decline in patients with COPD.^2^ Using a similar approach (disease probability measure), Ostridge et al^29^ generated an imaging marker of gas trapping as a surrogate for the presence of SAD that correlated significantly with R_5_ – R_19_ and Ax, oscillometric markers of SAD.

A description of other available methods to assess SAD, mainly used under research settings, such as hyperpolarized gas MRI and endobronchial optical coherence tomography, is beyond the scope of this review, focusing on everyday clinical practice, and the reader is referred to comprehensive reviews in the literature.^3^^,^^12^

Thus, the oscillometry indices of R_5_ – R_19_, R_5_ – R_20_, and Ax are specific and sensitive markers of SAD in patients with COPD that are available in daily clinical practice, whereas spirometry and body plethysmography may provide some indirect evidence for the presence of SAD. Nitrogen washout and functional CT imaging also may provide valuable information, although hitherto not widely available.

### Does SAD Assessment Contribute to Patient Diagnosis?

Oscillometric markers related to SAD, that is Ax, R_5_ – R_20_, and resonant frequency, have shown excellent accuracy in identifying patients with COPD (defined by an FEV_1_ to FVC ratio less than the lower limit of normal or < 0.7], compared with those who smoke and healthy participants.^30^ Moreover, in an older adult population in whom the forced expiration maneuver may be technically challenging, R_5_ – R_20_ showed excellent performance (area under the receiver operating characteristic curve, 0.883) for the diagnosis of COPD.^31^ The difference between inspiratory to expiratory reactance in oscillometry has been shown to discriminate between patients with asthma and patients with COPD^32^ and to support a COPD diagnosis in patients with contraindications or who are unable to perform spirometry.^33^ Intrabreath analysis of oscillometry also may be used to differentiate between interstitial and obstructive diseases.^34^ Indeed, the frequency dependence of resistance and reactance has been shown to be increased in patients with symptoms suggestive of COPD, even in those with preserved spirometry.^35^ This highlights the increased sensitivity of oscillometry and its value in the diagnosis of COPD. However, in the Evaluation of COPD Longitudinally to Identify Predictive Surrogate Endpoints (ECLIPSE) study, 40% and 29% of patients with COPD showed an R_5_ – R_20_ and an Ax value within the normal range, respectively, suggesting that assessment of SAD should not substitute spirometry for the diagnosis of COPD^6^ and supporting an integrative approach for lung function testing interpretation. In this study, measurements were performed after bronchodilation and using an impulse oscillometry device, shown to be inferior to modern devices in patients with COPD,^36^ and therefore raising the issue of reduced sensitivity. However, these findings are supported further by 2 recent large observational studies by Veneroni et al^37^ and Qvarnström et al,^38^ reporting that in a general population with respiratory symptoms, 26.8% and 10.5%, respectively, of patients showed abnormal oscillometry findings, despite normal spirometry findings, whereas 19% and 16.9%, respectively, showed abnormal spirometry findings, despite normal oscillometry findings. Although, in general, abnormal oscillometry findings in patients with preserved spirometry findings can be attributed to the increased sensitivity of oscillometry for peripheral airways dysfunction, reflecting different aspects of lung mechanics, the opposite, that is, normal oscillometry findings despite an abnormal spirometry findings, needs to be investigated further.

Longitudinal changes in both resistance and reactance follow the severity of airflow obstruction as defined by FEV_1_ decline.^39^ Given the updated recommendation to grade lung function impairment using cutoffs of the *z* score for FEV_1_, that is, –2.5 for mild disease and –4.0 for moderate disease,^11^ equivalent cutoff values of the R_5_ – R_20_ *z* score are 2.0 and 3.2, respectively, in patients with COPD, as estimated in a study using the impulse oscillometry technique.^40^ Thus, oscillometry has a complementary role not only for the diagnosis but also for the monitoring of lung function in COPD.

Although the persistence of airflow limitation after bronchodilation confirms the diagnosis of COPD,^41^ the clinical relevance of measuring the bronchodilator response, other than confirming the diagnosis of COPD, is under debate. Indeed, the current GOLD report does not recommend the evaluation of the degree of bronchodilator response to guide therapeutic decisions. However, recent findings based on the Genetic Epidemiology of Chronic Obstructive Pulmonary Disease (COPDGene) cohort suggest that reversibility after bronchodilation indeed may offer a phenotyping tool for patients with COPD, with differences in QoL and exacerbation frequency^42^ and an association with a more rapid decline in FEV_1_.^43^ Using a 30% threshold for positive increase in FEF_25%-75%_ after bronchodilation, Alobaidi et al^44^ detected an increased prevalence of small airways bronchodilator responsiveness (approximately 59% of patients) in COPD. Normal limits for an acute bronchodilator response have been reported for oscillometric parameters.^25^ Recently, Greig et al^45^ showed that oscillometry was more sensitive to detect a bronchodilator response to salbutamol, especially for Ax, a marker of small airways dysfunction, compared with spirometry (FEV_1_). Nevertheless, a systematic review concluded that heterogeneity exists in the literature regarding the physiologic markers used, the methodology, and the cutoff values used to define a positive bronchodilator response of small airways,^46^ and thus further research is needed.

Recently, pre-COPD, defined as the presence of symptoms, structural or physiologic abnormalities, or a combination thereof in the absence of obstructive spirometry findings has received increased attention.^47^ Using histopathologic analysis and micro-CT imaging of lung tissue samples, SAD has been detected in patients with pre-COPD.^48^ Given its increased sensitivity, oscillometry is found to detect SAD in symptomatic patients who smoke with preserved spirometry results.^35^ This finding has been reproduced in the recent Change in Airway Peripheral Tone in COPD (CAPTO-COPD) trial, in which SAD was detected in almost 60% of patients with mild COPD or symptomatic patients who smoked with preserved lung function through increased acinar zone inhomogeneity (multibreath nitrogen washout) or Ax (oscillometry). Indeed, oscillometric markers of SAD were increased in patients with respiratory symptoms but preserved spirometry findings, and resonant frequency outperformed FEF_25%-75%_ in the detection of SAD in this population.^49^ Again, in the ECLIPSE cohort, elevated Ax was measured in a subgroup of apparently healthy individuals who smoked and showed evidence of mild airflow limitation.^6^ Hence, detecting SAD enforces the recognition of pre-COPD or early COPD, an important milestone in COPD natural course.^28^

Thus, detection of small airways dysfunction, mainly by oscillometry, contributes to the diagnosis of COPD and functional severity grading, especially when spirometry is not feasible, and may be present even in patients with preserved spirometry results, although it should not substitute for spirometry or imaging, suggesting an integrational approach.

### Does SAD Assessment Contribute to Patient Outcomes?

The oscillometric index R_5_ – R_20_ has been shown to correlate more strongly with QoL and dyspnea (St. George’s Respiratory Questionnaire score and Medical Research Council dyspnea scale, respectively), compared to FEV_1_,^50^ and the presence of SAD (defined as R_5_ – R_20_ of ≥ 0.07 kPa/s/L) is an independent risk factor for increased symptoms (defined by a COPD Assessment Test score of ≥ 10).^5^ A major component of QoL impairment in patients with COPD is the reduction in exercise capacity and physical activity. In this regard, SAD and ventilation heterogeneity, assessed by nitrogen washout, independently was associated negatively with the 6-minute walking distance.^51^ Furthermore, the within-breath analysis of oscillometry (inspiratory minus expiratory reactance) and rapid tracking of changes in impedance during breathing (intrabreath analysis^52^) detects tidal airflow limitation^53^^,^^54^ that leads to dynamic hyperinflation, a major respiratory contributor of exercise limitation in COPD, and correlates with patient symptoms as assessed by the COPD Assessment Test and Clinical COPD Questionnaire scores.^55^ These data reveal an important link of SAD with patient-reported outcomes (symptoms, QoL), in accordance with recently published studies in other airway diseases, including asthma, cystic fibrosis, and bronchiectasis.^15^ This is supported further by a recent large study in which the presence of abnormal oscillometry findings (mainly reactance at 5 Hz) was associated significantly with respiratory symptoms, even in the presence of normal spirometry findings.^37^

Given the absence of lung function testing for future exacerbation risk assessment and therapeutic management in the current GOLD report,^41^ whether combining spirometry and small airways function (eg, by oscillometry) increases the predictive accuracy for COPD exacerbations is of interest. Interestingly, functional SAD defined by CT scan analysis (parametric response mapping) was an independent risk factor for future exacerbation in the Subpopulations and Intermediate Outcome Measures in COPD Study (SPIROMICS) cohort, with an OR of less than previous exacerbation history, but more than spirometric FEV_1_.^10^ Moreover, acinar zone inhomogeneity was found to be significantly increased in frequent (≥ 2/y) compared with infrequent exacerbators^56^ in a study in which the markers of SAD, R_5_ – R_19_ and residual volume to total lung capacity ratio, also correlated strongly with bronchoalveolar lavage neutrophils in those with frequent exacerbations. Finally, data from the UK Biobank cohort showed that small airways obstruction (defined as FEV_3_ to FEV_6_ ratio less than lower limit of normal) was associated with an increased risk of all-cause, cardiovascular, respiratory, and neoplasm mortality, suggesting SAD as a prognostic marker of mortality.^8^

Rapid loss of lung function, as defined by rapid FEV_1_ decline, is one of the main trajectories of the natural course of COPD.^57^ Interestingly, functional SAD detected by CT imaging (parametric response mapping analysis) independently predicts lung function decline,^2^ outperforming the effect of emphysema, especially in the early stages of disease, when the main loss of function is known to occur. The importance of SAD in driving disease progression is supported further by recently published results from the British Early COPD Network Cohort (BEACON) cohort, in which SAD detected by CT imaging (disease probability measure-defined air trapping), was associated independently with loss of FEV_1_ in young people who smoked and demonstrated normal spirometry findings.^58^

**Figure 2:**
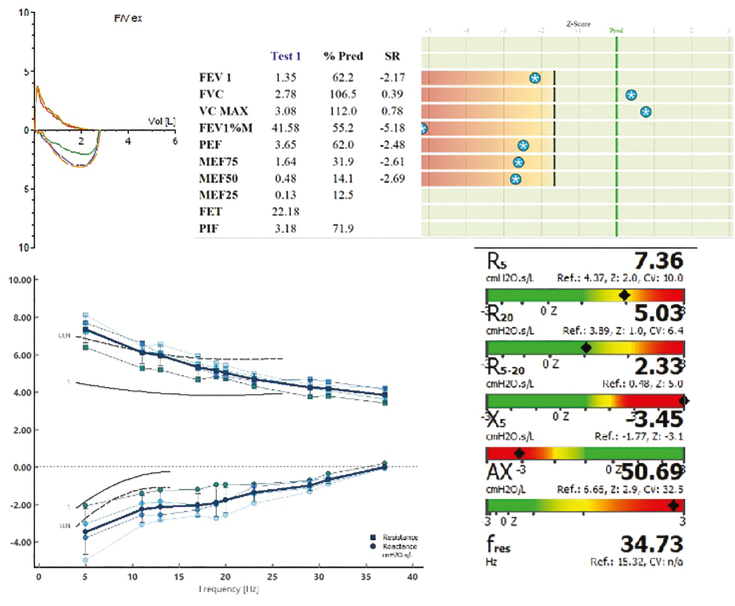
Image illustrating small airways assessment via oscillometry in a patient with COPD. A 70-year-old woman who formerly smoked, experienced infrequent exacerbations, and was receiving dual bronchodilator therapy underwent oscillometry as part of a re-evaluation for stable COPD. Graph showing increased frequency dependence of both resistance and reactance, resulting in increased R_5_ – R_20_ and Ax values, respectively, suggesting the presence of small airways dysfunction and ventilation inhomogeneity. CT scan analysis performed previously showed absence of emphysema, suggesting small airways disease as the main cause of ventilation heterogeneity. Note the out-proportional increased R_5_ – R_20_ (2.33 cm H_2_O/s/L; *z* = 5.0) compared with the FEV_1_ decrease (62% predicted; z = –2.17), supporting small airways disease as a major pathophysiologic cause of the patient’s lung function decline and increased symptom burden (COPD Assessment Test score, 20). Interestingly, reviewing the patient’s medical record revealed an accelerated decline of lung function with an approximately 110 mL/y loss of FEV_1_ over the previous 5 years. The potential therapeutical application of these finding is discussed in the text. Ax = area under the curve of reactance from the lowest frequency measured; F_res_ = resonant frequency; R = resistance; R_5_ = total resistance at 5 Hz; R_20_ = total resistance at 20 Hz; SR = standardized residual; VC = vital capacity; X = reactance; X_5_ = reactance at 5 Hz.

Finally, the assessment of small airways function can be used to monitor the physiologic response to long-acting bronchodilators. For example, a reduction of Ax from 2 hours after a long-acting bronchodilator inhalation correlated strongly with gas trapping reduction, as assessed by body plethysmography.^59^ Changes in resonant frequency, assessed by oscillometry, enabled the unmasking of an improved bronchodilator effect of tiotropium vs salmeterol in patients with moderate to severe COPD, despite similar increases in FEV_1_.^60^ Figure 2 illustrates the assessment of a patient with COPD and SAD. Thus, assessment of small airways dysfunction in patients with COPD correlates with disease outcomes, such as symptoms, exercise capacity, QoL, and exacerbations, and enables detection of therapeutic response to bronchodilators.

### Can We Treat SAD?

Several factors of inhaled therapy affect the deposition of inhaled drugs, including either the aerosol characteristics or patient factors, for instance, inhalation pattern.^61^ Of these, particle size seems to be the most important factor, with extra fine particles, that is, particles with a mass median aerodynamic diameter of < 2.1 μm, achieving a more peripheral deposition.^62^ In this regard, several in vitro and in vivo studies have shown that by modifying aerosol properties, small airways can be targeted.^63^ For example, administration of tiotropium in patients with COPD using a dry powder inhaler failed to improve ventilation inhomogeneity in the acinar (peripheral) zone,^64^ whereas when using a soft mist inhaler that improves peripheral deposition, tiotropium clearly improved small airways function, as assessed by oscillometry.^65^

In recent years, advances in the field have been made using functional respiratory imaging, whereby combining anatomic reconstructions of the respiratory system though CT scans and superimposing flow-simulation (computational fluid dynamics), the regional deposition of inhaled drugs can be estimated in a lung model.^66^ Using this technology, Usmani et al^67^ showed that an extra fine formulation of beclomethasone, formoterol, and glycopyrronium achieved a higher peripheral distribution and airway penetration of all 3 drug components compared with a non-extra fine formulation of fluticasone furoate, vilanterol, and umeclidinium in patients with moderate to severe COPD. Most importantly, the extra fine formulation achieved an almost 3-fold increase in peripheral distribution of the inhaled corticosteroid component, thus directing anti-inflammatory therapy to the small airways, where active inflammatory processes occur. These findings were reproduced by a subsequent study using the functional respiratory imaging technique in patients with COPD, in which a variety of inhalation profiles also were modelled (inhalation duration, flow, or both), to mimic real-life settings, where an optimal inhalation technique may not be achieved.^68^ Similarly, using functional respiratory imaging and γ scintigraphy, an established method to assess regional drug deposition, increased peripheral deposition was found for the combination of budesonide, glycopyrrolate, and formoterol using a novel cosuspension delivery technology in patients with COPD,^69^ with similar deposition patterns, regardless of disease stage (moderate vs severe).^70^ Interestingly, administration of an extra fine particle formulation of beclomethasone and formoterol in patients with severe COPD resulted in improvement of small airways function, as assessed by R_5_ – R_20_, and this correlated positively with improvement in symptoms, as assessed by the COPD Assessment Test score.^71^

Clinical data on the comparison between inhaled therapies targeting small airways (eg, extra fine formulations) vs other formulations are limited. Previously, Tzani et al^72^ showed that in a selected group of patients with COPD and hyperinflation (FEV_1_ < 65% and functional residual capacity > 120% predicted), an extra fine particle formulation of beclomethasone and formoterol administered via a metered-dose inhaler was more efficient in reducing gas trapping and improving dyspnea compared with fluticasone and salmeterol via a dry powder inhaler. Similarly, a head-to-head comparison of extra fine beclomethasone and formoterol via metered-dose inhaler with fluticasone and salmeterol via dry powder inhaler in patients with COPD showed an improved dyspnea score for the extra fine formulation.^73^ However, both trials were small to enable a robust conclusion and investigated an inhaled corticosteroid plus long-acting β2 agonist combination that now is not encouraged for COPD therapy unless a concomitant asthma diagnosis is present. Recently, triple therapy with an extra fine formulation of beclomethasone, formoterol, and glycopyrronium further improved small airways function compared with extra fine beclomethasone and formoterol in a prespecified group of patients with COPD with hyperinflation.^74^

Finally, although currently no therapeutic interventions are available for pre-COPD,^47^ early identification of SAD, possibly at a stage when lesions are reversible, may motivate those who smoke toward smoking cessation and may foster preventive strategies, thus constituting an indirect therapeutic potential of actively assessing SAD. Indeed, smoking cessation results in improvement of phase 3 slope of single breath nitrogen washout and closing volume, attributed to reversal of peripheral airway obstruction.^75^

Thus, our therapeutic armamentarium includes inhaled drugs that specifically can target small airways in patients with COPD, for example, extra fine particles; however, we are in need of large trials that will show whether smaller particle size changes outcomes in people with SAD or if the presence of SAD predicts a different response to drugs like long-acting bronchodilators than in those with comparable FEV_1_ but no SAD.

## Future Directions

### What Are the Unmet Needs to Demystify Small Airways Further?

Over the last decades, increased attention has been attributed to the early origins of lung diseases, including COPD.^28^ Given the development of peripheral airways late into gestation and the continuous growth into early life, the role of SAD after early-life events warrants further research. Moreover, given that inflammation in the small airways seems to drive disease progression, the association of SAD with systemic inflammation in COPD and comorbidities merits further investigation.

Despite the progress in standardizing small airways function assessments, further research also is needed to harmonize measurements across different centers and to provide robust reference equations. Moreover, the effect of systemic therapies, for example, oral roflumilast or biologic therapies, that may enable targeting the peripheral lung through the systemic circulation should be investigated further. Finally, more clinical trials are needed that include markers of SAD as outcomes and therapies that target the small airways as treatment arms.

## Summary

**Figure 3:**
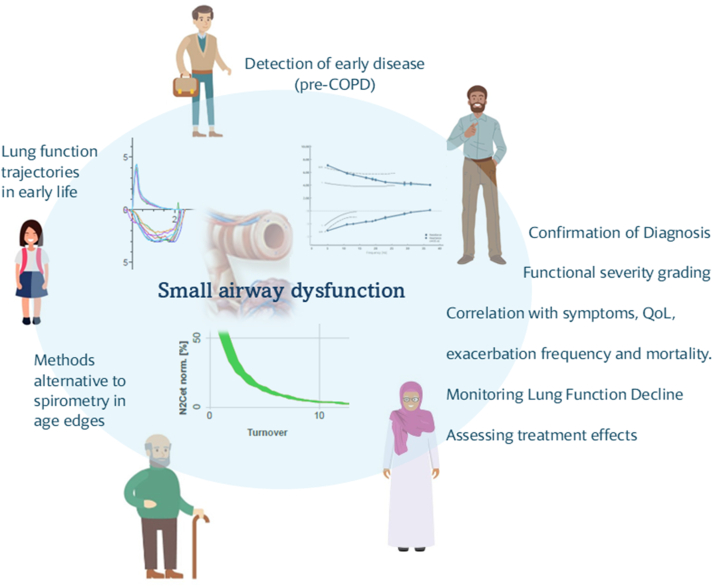
Proposed scheme for incorporating small airways function assessment in daily clinical practice across the natural course of COPD. Major risk factors, such as smoking exposure and air pollution, lead to small airways inflammation, obstruction and disease, early manifestations of COPD, and major contributors of disease progression. Assessment of small airways dysfunction with sensitive techniques (eg, oscillometry, nitrogen washout) is shown to contribute to detection of early disease. Detection of small airways dysfunction also contributes to diagnosis confirmation, functional severity grading, and monitoring of lung function decline. Moreover, small airways dysfunction correlates with disease outcomes, such as symptoms, QoL, and exacerbations. In this regard, using markers of small airways disease in treatment algorithms and targeting inhaled therapy in small airways may offer a unique opportunity for halting disease progression and improving patient outcomes. Finally, oscillometry may substitute spirometry in specific patient populations, such as older adults or children. N2Cet norm = normalized end tidal N2 concentration; QoL = quality of life.

As illustrated in Figure 3, accumulated evidence of the role of small airways assessment for the early identification, diagnosis, and monitoring of COPD, as well as its association with patient-reported outcomes and QoL, warrants its inclusion in the management plan of patients with COPD in daily clinical practice.

## Funding/Support

The authors have reported to *CHEST* that no funding was received for this study.

## Financial/Nonfinancial Disclosures

The authors have reported to *CHEST* the following: D. T. reports honoraria from Menarini, Chiesi, AstraZeneca, and GlaxoSmithKline outside the submitted work. O. S. U. reports grants and personal fees from AstraZeneca, Boehringer Ingelheim, Chiesi, GlaxoSmithKline, Cipla, and Mereo Biopharma and honoraria from AstraZeneca, Boehringer Ingelheim, Chiesi, GlaxoSmithKline, Mundi Pharma, Sandoz, Takeda, Cipla, Covis, Novartis, Orion, Menarini, UCB, Trudell Medical, Deva, and Kamada outside the submitted work. None declared (Y. K.).

## Supplementary Data

e-Online Data

## References

[1] Hogg J.C., Macklem P.T., Thurlbeck W.M. (1968). Site and nature of airway obstruction in chronic obstructive lung disease. N Engl J Med.

[2] Bhatt S.P., Soler X., Wang X. (2016). Association between functional small airway disease and FEV1 decline in chronic obstructive pulmonary disease. Am J Respir Crit Care Med.

[3] Usmani O.S., Han M.K., Kaminsky D.A. (2021). Seven pillars of small airways disease in asthma and COPD: supporting opportunities for novel therapies. Chest.

[4] Xiao D., Chen Z., Wu S. (2020). Prevalence and risk factors of small airway dysfunction, and association with smoking, in China: findings from a national cross-sectional study. Lancet Respir Med.

[5] Crisafulli E., Pisi R., Aiello M. (2017). Prevalence of small-airway dysfunction among COPD patients with different GOLD stages and its role in the impact of disease. Respiration.

[6] Crim C., Celli B., Edwards L.D. (2011). Respiratory system impedance with impulse oscillometry in healthy and COPD subjects: ECLIPSE baseline results. Respir Med.

[7] Knox-Brown B., Patel J., Potts J. (2023). Small airways obstruction and its risk factors in the Burden of Obstructive Lung Disease (BOLD) study: a multinational cross-sectional study. Lancet Glob Health.

[8] Quintero Santofimio V., Knox-Brown B., Potts J., Bartlett-Pestell S., Feary J., Amaral A.F.S. (2024). Small airways obstruction and mortality: findings from the UK Biobank. Chest.

[9] Dilektasli A.G., Porszasz J., Casaburi R. (2016). A novel spirometric measure identifies mild COPD unidentified by standard criteria. Chest.

[10] Han M.K., Quibrera P.M., Carretta E.E. (2017). Frequency of exacerbations in patients with chronic obstructive pulmonary disease: an analysis of the SPIROMICS cohort. Lancet Respir Med.

[11] Stanojevic S., Kaminsky D.A., Miller M.R. (2022). ERS/ATS technical standard on interpretive strategies for routine lung function tests. Eur Respir J.

[12] McNulty W., Usmani O.S. (2014). Techniques of assessing small airways dysfunction. Eur Clin Respir J.

[13] Quanjer P.H., Weiner D.J., Pretto J.J., Brazzale D.J., Boros P.W. (2014). Measurement of FEF25-75% and FEF75% does not contribute to clinical decision making. Eur Respir J.

[14] Yee N., Markovic D., Buhr R.G. (2022). Significance of FEV(3)/FEV(6) in recognition of early airway disease in smokers at risk of development of COPD: analysis of the SPIROMICS cohort. Chest.

[15] Kaminsky D.A., Simpson S.J., Berger K.I. (2022). Clinical significance and applications of oscillometry. Eur Respir Rev.

[16] King G.G., Bates J., Berger K.I. (2020). Technical standards for respiratory oscillometry. Eur Respir J.

[17] Bates J.H.T. (2009).

[18] Peters U., Kaminsky D.A., Bhatawadekar S., Lundblad L., Maksym G.N., Ionescu C. (2019). Lung Function Testing in the 21st Century.

[19] Deprato A., Ferrara G., Bhutani M. (2022). Reference equations for oscillometry and their differences among populations: a systematic scoping review. Eur Respir Rev.

[20] Dandurand R.J., Lavoie J.P., Lands L.C., Hantos Z., Oscillometry Harmonisation Study Group (2019). Comparison of oscillometry devices using active mechanical test loads. ERJ Open Res.

[21] Lipworth B.J., Jabbal S. (2018). What can we learn about COPD from impulse oscillometry?. Respir Med.

[22] Otis A.B., McKerrow C.B., Bartlett R.A. (1956). Mechanical factors in distribution of pulmonary ventilation. J Appl Physiol.

[23] Milne S., Jetmalani K., Chapman D.G. (2019). Respiratory system reactance reflects communicating lung volume in chronic obstructive pulmonary disease. J Appl Physiol.

[24] Eddy R.L., Westcott A., Maksym G.N., Parraga G., Dandurand R.J. (2019). Oscillometry and pulmonary magnetic resonance imaging in asthma and COPD. Physiol Rep.

[25] Oostveen E., Boda K., van der Grinten C.P. (2013). Respiratory impedance in healthy subjects: baseline values and bronchodilator response. Eur Respir J.

[26] Cosio M., Ghezzo H., Hogg J.C. (1978). The relations between structural changes in small airways and pulmonary-function tests. N Engl J Med.

[27] Verbanck S., Schuermans D., Meysman M., Paiva M., Vincken W. (2004). Noninvasive assessment of airway alterations in smokers: the small airways revisited. Am J Respir Crit Care Med.

[28] Stolz D., Mkorombindo T., Schumann D.M. (2022). Towards the elimination of chronic obstructive pulmonary disease: a Lancet Commission. Lancet.

[29] Ostridge K., Gove K., Paas K.H.W. (2019). Using novel computed tomography analysis to describe the contribution and distribution of emphysema and small airways disease in chronic obstructive pulmonary disease. Ann Am Thorac Soc.

[30] Chaiwong W., Namwongprom S., Liwsrisakun C., Pothirat C. (2020). Diagnostic ability of impulse oscillometry in diagnosis of chronic obstructive pulmonary disease. COPD.

[31] Liu Z., Lin L., Liu X. (2017). Clinical application value of impulse oscillometry in geriatric patients with COPD. Int J Chron Obstruct Pulmon Dis.

[32] Paredi P., Goldman M., Alamen A. (2010). Comparison of inspiratory and expiratory resistance and reactance in patients with asthma and chronic obstructive pulmonary disease. Thorax.

[33] Singh P., Ahuja N.B., Krishna S.V.S. (2025). Role of oscillometry to diagnose obstructive airway diseases in patients who are unable to perform spirometry correctly. Medical Journal Armed Forces India.

[34] Wu J.K.Y., Ma J., Nguyen L. (2022). Correlation of respiratory oscillometry with CT image analysis in a prospective cohort of idiopathic pulmonary fibrosis. BMJ Open Respir Res.

[35] Frantz S., Nihlen U., Dencker M., Engstrom G., Lofdahl C.G., Wollmer P. (2012). Impulse oscillometry may be of value in detecting early manifestations of COPD. Respir Med.

[36] Lundblad L.K.A., Miletic R., Piitulainen E., Wollmer P. (2019). Oscillometry in chronic obstructive lung disease: in vitro and in vivo evaluation of the impulse oscillometry and tremoflo devices. Sci Rep.

[37] Veneroni C., Valach C., Wouters E.F.M. (2024). Diagnostic potential of oscillometry: a population-based approach. Am J Respir Crit Care Med.

[38] Qvarnström B., Engstrom G., Frantz S. (2023). Impulse oscillometry indices in relation to respiratory symptoms and spirometry in the Swedish Cardiopulmonary Bioimage Study. ERJ Open Res.

[39] Di Mango A.M., Lopes A.J., Jansen J.M., Melo P.L. (2006). Changes in respiratory mechanics with increasing degrees of airway obstruction in COPD: detection by forced oscillation technique. Respir Med.

[40] Liang X., Zheng J., Gao Y. (2022). Clinical application of oscillometry in respiratory diseases: an impulse oscillometry registry. ERJ Open Res.

[41] Global Initiative for Chronic Obstructive Lung Disease 2024 GOLD Report. Global Initiative for Chronic Obstructive Lung Disease website. https://goldcopd.org/2024-gold-report/.

[42] Hansen J.E., Dilektasli A.G., Porszasz J. (2019). A new bronchodilator response grading strategy identifies distinct patient populations. Ann Am Thorac Soc.

[43] Fortis S., Quibrera P.M., Comellas A.P. (2023). Bronchodilator responsiveness in tobacco-exposed people with or without COPD. Chest.

[44] Alobaidi N.Y., Almeshari M.A., Stockley J.A., Stockley R.A., Sapey E. (2022). The prevalence of bronchodilator responsiveness of the small airway (using mid-maximal expiratory flow) in COPD—a retrospective study. BMC Pulm Med.

[45] Greig R., Kuo C.R., Chan R., Lipworth B. (2025). Reversibility of airwave oscillometry in COPD. Respir Med.

[46] Almeshari M.A., Alobaidi N.Y., Sapey E., Usmani O., Stockley R.A., Stockley J.A. (2021). Small airways response to bronchodilators in adults with asthma or COPD: a systematic review. Int J Chron Obstruct Pulmon Dis.

[47] Martinez F.J., Agusti A., Celli B.R. (2022). Treatment trials in young patients with chronic obstructive pulmonary disease and pre-chronic obstructive pulmonary disease patients: time to move forward. Am J Respir Crit Care Med.

[48] Verleden S.E., Hendriks J.M.H., Snoeckx A. (2024). Small airway disease in pre-chronic obstructive pulmonary disease with emphysema: a cross-sectional study. Am J Respir Crit Care Med.

[49] Chiu H.Y., Hsiao Y.H., Su K.C., Lee Y.C., Ko H.K., Perng D.W. (2020). Small airway dysfunction by impulse oscillometry in symptomatic patients with preserved pulmonary function. J Allergy Clin Immunol Pract.

[50] Haruna A., Oga T., Muro S. (2010). Relationship between peripheral airway function and patient-reported outcomes in COPD: a cross-sectional study. BMC Pulm Med.

[51] Lopes A.J., Mafort T.T. (2014). Correlations between small airway function, ventilation distribution, and functional exercise capacity in COPD patients. Lung.

[52] Hantos Z. (2021). Intra-breath oscillometry for assessing respiratory outcomes. Curr Opin Physiol.

[53] Dellaca R.L., Santus P., Aliverti A. (2004). Detection of expiratory flow limitation in COPD using the forced oscillation technique. Eur Respir J.

[54] Lorx A., Czovek D., Gingl Z. (2017). Airway dynamics in COPD patients by within-breath impedance tracking: effects of continuous positive airway pressure. Eur Respir J.

[55] Nasr A., Papapostolou G., Jarenback L. (2024). Expiratory and inspiratory resistance and reactance from respiratory oscillometry defining expiratory flow limitation in obstructive lung diseases. Clin Physiol Funct Imaging.

[56] Day K., Ostridge K., Conway J. (2021). Interrelationships among small airways dysfunction, neutrophilic inflammation, and exacerbation frequency in COPD. Chest.

[57] Lange P., Celli B., Agusti A. (2015). Lung-function trajectories leading to chronic obstructive pulmonary disease. N Engl J Med.

[58] Ritchie A.I., Donaldson G.C., Hoffman E.A. (2024). Structural predictors of lung function decline in young smokers with normal spirometry. Am J Respir Crit Care Med.

[59] Milne S., Hammans C., Watson S., Farah C.S., Thamrin C., King G.G. (2018). Bronchodilator responses in respiratory impedance, hyperinflation and gas trapping in COPD. COPD.

[60] Borrill Z.L., Houghton C.M., Tal-Singer R. (2008). The use of plethysmography and oscillometry to compare long-acting bronchodilators in patients with COPD. Br J Clin Pharmacol.

[61] Usmani O.S. (2012). Treating the small airways. Respiration.

[62] Usmani O.S., Biddiscombe M.F., Barnes P.J. (2005). Regional lung deposition and bronchodilator response as a function of beta2-agonist particle size. Am J Respir Crit Care Med.

[63] Usmani O.S., Dhand R., Lavorini F., Price D. (2021). Why we should target small airways disease in our management of chronic obstructive pulmonary disease. Mayo Clin Proc.

[64] Verbanck S., Schuermans D., Vincken W. (2007). Small airways ventilation heterogeneity and hyperinflation in COPD: response to tiotropium bromide. Int J Chron Obstruct Pulmon Dis.

[65] Biddiscombe M., Saleem A., Meah S., Mak V., Usmani O. (2018). Efficacy of the device in targeting tiotropium to the small airways in COPD. Eur Respir J.

[66] Hajian B., De Backer J., Vos W., Van Holsbeke C., Clukers J., De Backer W. (2016). Functional respiratory imaging (FRI) for optimizing therapy development and patient care. Expert Rev Respir Med.

[67] Usmani O.S., Scichilone N., Mignot B. (2020). Airway deposition of extrafine inhaled triple therapy in patients with COPD: a model approach based on functional respiratory imaging computer simulations. Int J Chron Obstruct Pulmon Dis.

[68] Usmani O., Li G., De Backer J., Sadafi H., Wu L., Marshall J. (2023). Modeled small airways lung deposition of two fixed-dose triple therapy combinations assessed with in silico functional respiratory imaging. Respir Res.

[69] Usmani O.S., Roche N., Jenkins M., Stjepanovic N., Mack P., De Backer W. (2021). Consistent pulmonary drug delivery with whole lung deposition using the aerosphere inhaler: a review of the evidence. Int J Chron Obstruct Pulmon Dis.

[70] Usmani O., Roche N., Wahab E. (2021). A scintigraphy study of budesonide/glycopyrrolate/formoterol fumarate metered dose inhaler in patients with moderate-to-very severe chronic obstructive pulmonary disease. Respir Res.

[71] Pisi R., Aiello M., Piraino A. (2021). Beclomethasone/formoterol in extra-fine formulation improves small airway dysfunction in COPD patients. Pulm Ther.

[72] Tzani P., Crisafulli E., Nicolini G. (2011). Effects of beclomethasone/formoterol fixed combination on lung hyperinflation and dyspnea in COPD patients. Int J Chron Obstruct Pulmon Dis.

[73] Singh D., Nicolini G., Bindi E. (2014). Extrafine beclomethasone/formoterol compared to fluticasone/salmeterol combination therapy in COPD. BMC Pulm Med.

[74] Dean J., Panainte C., Khan N., Singh D. (2020). The TRIFLOW study: a randomised, cross-over study evaluating the effects of extrafine beclometasone/formoterol/glycopyrronium on gas trapping in COPD. Respir Res.

[75] Bode F.R., Dosman J., Martin R.R., Macklem P.T. (1975). Reversibility of pulmonary function abnormalities in smokers. A prospective study of early diagnostic tests of small airways disease. Am J Med.

