# Supplementary material for: Small Airways Disease in Patients With COPD: A Question-and-Answer Approach for Everyday Clinical Practice
Source: Chest. 2025 Jul 16;169(3):641–51. doi: 10.1016/j.chest.2025.07.002 (PMC12975394; doi:10.1016/j.chest.2025.07.002)
Supplement: e-Online Data [file mmc1.docx]

**Online data supplement**

**Small airway disease in COPD patients: A Q&A approach for everyday clinical practice.**

Dimitrios Toumpanakis, Youlim Kim, Omar S Usmani

**LITERATURE SEARCH**

We searched articles on PubMed and Google Scholar, with the following keywords, small airways AND COPD plus techniques or oscillometry or nitrogen washout or imaging or symptoms or patient reported outcomes or bronchodilators or particle deposition. The first literature search was completed by November 2024, whereas another search was performed in May 2025, during the revision of the manuscript to avoid missing significant progress in the field. Based on the experience of the authors, we focused on studies exploring the clinical usefulness of small airway assessment in COPD. Reference lists of included studies were also reviewed to identify additional relevant studies. Although our literature search was not limited to a specific time-period, we focused on recently published, original research studies. Only studies written in English were included.

**What are the** **immunopathological features of small airways disease?**

Following the seminal work by Hogg et al, in 1968 that established the small airways as the main site of increased resistance in COPD^1^ and the recognition that spirometry “misses” structural changes of the small airways detected by more sensitive physiological techniques (e.g. nitrogen washout),^2^ several studies in the last two decades have delineated the nature and pathophysiological consequences of small airway disease in COPD. Thickening of the small airway wall from remodelling, infiltration from innate and adaptive immunity cells, lymphoid follicles and accumulation of intraluminal mucus have been described in COPD,^3^ whereas loss of intra-alveolar attachments of small airways is the histological lesion more strongly linked to severity of airflow limitation.^4^

Recently Booth et al, showed that M1-like macrophages and neutrophil sequestration, together with associated gene signatures, were associated with elastin fiber reduction in alveolar attachments leading to their loss, while innate immune cells in small airways were linked to airway wall remodelling.^5^ In this study, loss of alveolar attachments was apparent irrespectively of parenchymal emphysematous destruction, corroborating previous studies showing SAD precedes emphysema formation.^6^

**REFERENCES**

E1. Hogg JC, Macklem PT, Thurlbeck WM. Site and nature of airway obstruction in chronic obstructive lung disease. *N Engl J Med.* 1968;278(25):1355-1360.

E2. Cosio M, Ghezzo H, Hogg JC, et al. The relations between structural changes in small airways and pulmonary-function tests. *N Engl J Med.* 1978;298(23):1277-1281.

E3. Hogg JC, Chu F, Utokaparch S, et al. The nature of small-airway obstruction in chronic obstructive pulmonary disease. *N Engl J Med.* 2004;350(26):2645-2653.

E4. Polosukhin VV, Gutor SS, Du RH, et al. Small airway determinants of airflow limitation in chronic obstructive pulmonary disease. *Thorax.* 2021;76(11):1079-1088.

E5. Booth S, Hsieh A, Mostaco-Guidolin L, et al. A Single-Cell Atlas of Small Airway Disease in Chronic Obstructive Pulmonary Disease: A Cross-Sectional Study. *Am J Respir Crit Care Med.* 2023;208(4):472-486.

E6. McDonough JE, Yuan R, Suzuki M, et al. Small-airway obstruction and emphysema in chronic obstructive pulmonary disease. *N Engl J Med.* 2011;365(17):1567-1575.
